# Molecular Detection and Genotyping of Japanese Encephalitis Virus in Mosquitoes during a 2010 Outbreak in the Republic of Korea

**DOI:** 10.1371/journal.pone.0055165

**Published:** 2013-02-04

**Authors:** Hyun-Ji Seo, Heung Chul Kim, Terry A. Klein, Andrew M. Ramey, Ji-Hye Lee, Soon-Goo Kyung, Jee-Yong Park, Yun Sang Cho, In-Soo Cho, Jung-Yong Yeh

**Affiliations:** 1 Foreign Animal Disease Division, Animal, Plant & Fisheries Quarantine & Inspection Agency, Anyang-si, Republic of Korea; 2 5th Medical Detachment, 168th Multifunctional Medical Battalion, 65th Medical Brigade, Unit 15247, Seoul, Republic of Korea; 3 Force Health Protection and Preventive Medicine, 65th Medical Brigade/USAMEDDAC-Korea, Unit 15281, Seoul, Republic of Korea; 4 US Geological Survey, Alaska Science Center, Anchorage, Arkansas, United States of America; 5 Korea Racing Authority, Gwacheon, Republic of Korea; 6 Division of Life Sciences, College of Life Sciences and Bioengineering, University of Incheon, Incheon, Republic of Korea; Duke-National University of Singapore Graduate Medical School, Singapore

## Abstract

Japanese encephalitis virus (JEV), a mosquito-borne zoonotic pathogen, is one of the major causes of viral encephalitis. To reduce the impact of Japanese encephalitis among children in the Republic of Korea (ROK), the government established a mandatory vaccination program in 1967. Through the efforts of this program only 0–7 (mean 2.1) cases of Japanese encephalitis were reported annually in the ROK during the period of 1984–2009. However, in 2010 there was an outbreak of 26 confirmed cases of Japanese encephalitis, including 7 deaths. This represented a >12-fold increase in the number of confirmed cases of Japanese encephalitis in the ROK as compared to the mean number reported over the last 26 years and a 3.7-fold increase over the highest annual number of cases during this same period (7 cases). Surveillance of adult mosquitoes was conducted during the 2010 outbreak of Japanese encephalitis in the ROK. A total of 6,328 culicine mosquitoes belonging to 12 species from 5 genera were collected at 6 survey sites from June through October 2010 and assayed by reverse-transcription polymerase chain reaction (RT-PCR) for the presence of JEV. A total of 34/371 pooled samples tested positive for JEV (29/121 *Culex tritaeniorhynchus*, 4/64 *Cx. pipiens*, and 1/26 *Cx. bitaeniorhynchus*) as confirmed by sequencing of the pre-membrane and envelope protein coding genes. The maximum likelihood estimates of JEV positive individuals per 1,000 culicine vectors for *Cx. tritaeniorhynchus*, *Cx. pipiens*, and *Cx. bitaeniorhynchus* were 11.8, 5.6, and 2.8, respectively. Sequences of the JEV pre-membrane and envelope protein coding genes amplified from the culicine mosquitoes by RT-PCR were compared with those of JEV genotypes I-V. Phylogenetic analyses support the detection of a single genotype (I) among samples collected from the ROK in 2010.

## Introduction

Japanese encephalitis virus (JEV), the prototype member of the JEV serocomplex within the genus *Flavivirus*, family *Flaviviridae*, is a single stranded positive sense RNA virus. The genome of JEV is approximately 11,000 base pairs (bp) in length and contains of 3 structural proteins (capsid, membrane, and envelope proteins) and 7 nonstructural proteins (NS1, NS2a, NS2b, NS3, NS4a, NS4b, and NS5) [1]–[3]. JEV is one of the major causes of viral encephalitis worldwide and the most significant arthropod-borne viral encephalitis causing agent in east and southeast Asia [4]. An estimated three billion persons live in JEV-endemic countries [5], and the annual incidence of Japanese encephalitis (JE) is 30,000–50,000 cases [6]. The global economic and human health impacts of JE are impressive with 10,000–15,000 deaths attributed to this disease annually and an estimated 709,000 disability-adjusted life years reported for 2002 [6], [7].

JEV is transmitted principally by rice paddy-breeding *Culex* mosquitoes in an enzootic cycle involving an avian reservoir and porcine (domestic and feral) amplifying hosts. Humans and other non-avian vertebrates (e.g., horses) are only infected with JEV incidentally and are considered “dead-end hosts” because they usually fail to produce viremia of sufficient titer to infect mosquitoes. The prototype JEV strain was isolated in Japan in 1935 [8], and the virus has since been found throughout east and southeast Asia, with the geographical borders of viral activity extending north to maritime Siberia [9], west to Pakistan [10], southeast to Australia [11], and northeast to Japan and the Korean Peninsula [12]. JEV strains are divided into five genotypes distributed throughout this geographical range. China is a highly epidemic area of JE activity [13] and more than 100 JEV strains belonging to genotypes I, III, and V have been isolated from different hosts in this country since the 1950s [2]. JEV genotype III was predominant in China prior to 2001; however, since the first detection of a genotype I virus in this country (isolated in 1979), the detection of this genotype has become increasingly common [14]. JEV genotype I viruses have recently been isolated from mosquitoes, swine, and humans in China and suggest that this virus type may become the predominant genotype in this country [1]–[3], [14], [15]. JEV genotype III strains were most common in the nearby Republic of Korea (ROK) prior to 1993. Genotypes I and III were both detected in ROK in 1994 and since then, only genotype I has been isolated [12].

**Figure 1:**
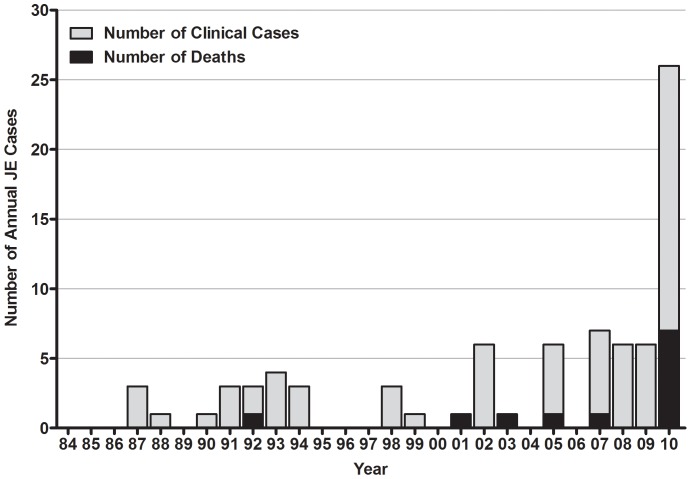
Clinical cases and deaths of Japanese encephalitis in Republic of Korea, 1984–2010. Figure is based on data provided by the Korea Center for Disease Control and Prevention, 2011.

In order to reduce the impact of JE among children in ROK, the government established a mandatory JEV vaccination program in 1967 that was expanded annually until all school-age children were included by 1971. Live attenuated JEV vaccine (SA14-14-2 strain) developed in China, as well as inactivated vaccine produced by formalin treatment of Nakayama strain virus cultivated in specific pathogen-free mouse brain, have been used in this vaccination program [16], [17]. Through the efforts of the vaccination program in ROK, only 0–7 cases (mean 2.1) of JE were reported annually during the period of 1984–2009 [18]. However, during 2010, there was an outbreak of 26 confirmed cases of JE including 7 deaths. This represented a >12-fold increase in the number of confirmed cases of Japanese encephalitis in ROK as compared to the mean reported over the last 26 years (55 cases including 5 deaths during 1984–2009) and a 3.7-fold increase over the highest annual number of cases (7 cases) [19] (Fig. 1).

Monitoring for the presence of JEV in mosquitoes can be used to estimate levels of potential JEV exposure, intensity of viral activity, and genetic variation of JEV throughout surveyed areas. Although the prevalence of JEV in mosquitoes has been previously reported in ROK, sampling efforts have been focused at or near United States (US) military installations and training sites [20], [21]. Thus, information on the nationwide prevalence of JEV in mosquitoes in ROK is limited [22]. The objectives of this study were to identify JEV genotypes circulating in ROK during the outbreak of JE in 2010, investigate the genetic variation and relative prevalence of virus strains, and identify mosquito species potentially involved in the transmission of JEV.

## Materials and Methods

### Survey Area and Mosquito Collection

**Figure 2:**
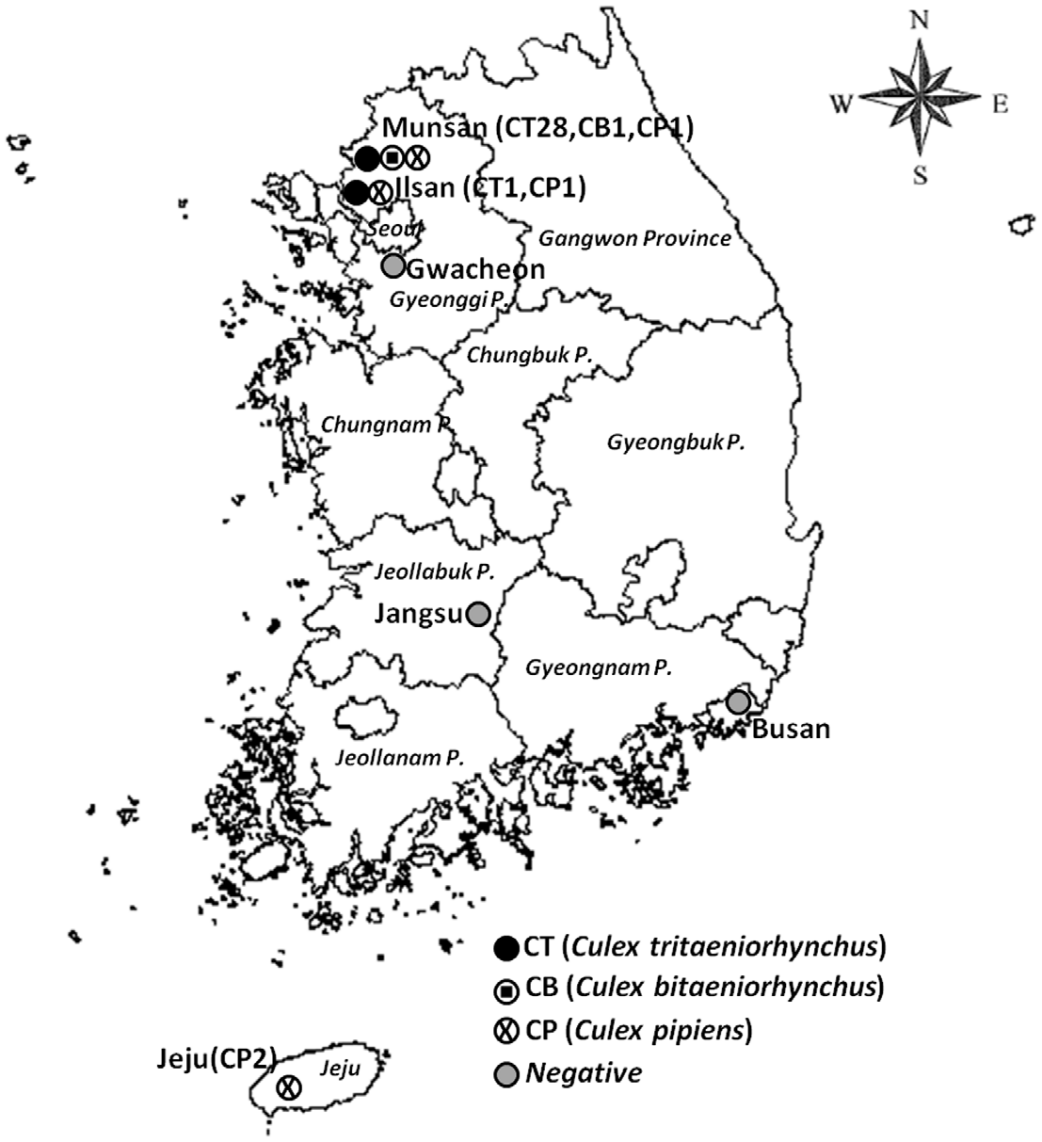
Collection sites and of Japanese encephalitis virus-positive pools, Republic of Korea, 2010. Abbreviations in parentheses indicate the number of Japanese encephalitis virus-positive pools by mosquito species.

As part of a national vector surveillance program for arboviral infectious diseases, adult mosquitoes were collected using a Mosquito Magnet (Pro-Model, American Biophysics Corp., Greenwich, RI, USA) at selected sites throughout ROK from April through October 2010. The Foreign Animal Disease Division, Animal, Plant, and Fisheries Quarantine and Inspection Agency (QIA, Anyang, ROK), and the 5^th^ Medical Detachment, 168^th^ Multifunctional Medical Battalion, 65^th^ Medical Brigade, collected mosquitoes biweekly at 6 survey sites in ROK: Munsan, Ilsan, Gwacheon, Jangsu, Busan, and Jeju Island (Fig. 2). The Ilsan, Gwacheon, Jangsu, Busan, and Jeju collection sites were at horse farms, whereas the Munsan collection site was at Warrior Base, a US military training site surrounded by rice paddies near the demilitarized zone (DMZ). All necessary permits were obtained for the described field studies from QIA, ROK Racing Authority, ROK Army, and the US Army. Up to 30 specimens of culicine mosquitoes were pooled in a 2 mL cryovials (Nalge Nunc International, NY, USA) according to species and date of collection, packaged with dry ice, and sent to the QIA where they were assayed for JEV.

### Reverse-Transcription Polymerase Chain Reaction, Genetic Sequencing, Assessment of Nucleotide Sequence Similarity, and Phylogenetic Analyses

Mosquito samples were homogenized in the laboratory and clarified by centrifugation. Total RNA was extracted from mosquito homogenate using a BioRobot M48 workstation apparatus (Qiagen, GmBH, Hilden, Germany) with a MagAttract Virus Mini M48 kit, (Qiagen). Nucleic acids were eluted in 50 µL of buffer and stored at −70°C.

RNA was assayed by reverse transcriptase polymerase chain reaction (RT-PCR) to detect JEV by targeting pre-membrane protein (prM) and envelope protein (E) coding genes using the Maxime™ RT PreMix (Intron Biotechnology, Seoungnam-si, Gyeonggi-do, ROK). The polymerase chain reaction (PCR) contained 2 µL of prepared cDNA and 10 pmol of each primer (JEE F/JEE R and JEV-prMF/JEV-prMR for reactions targeting the coding regions of the E and prM proteins, respectively) [14], [23] in Maxime PCR PreMix (Intron Biotechnology). Amplified products were visualized by electrophoresis on a 1.2% agarose gel stained with ethidium bromide (0.5 µg/mL) using 1x TAE migration buffer (pH 8.0; 40 mmol/L Tris-acetate, 1 mmol/L EDTA). Target JEV prM and E products were 674 and 1,541 bp, respectively.

The number of JEV positive mosquitoes per 1,000 individuals was estimated from assay results using maximum likelihood estimation. Maximum likelihood estimation takes into account the number of pooled samples, number of positive pooled samples, and variation in pooled sample size thereby relaxing the assumption of the minimum field infection rate that only one infected mosquito exists in a positive pooled sample. Maximum likelihood estimation may therefore be a more accurate measure of infection rate [24]–[25]. Maximum likelihood estimates (MLE) were calculated using PooledInfRate software [26].

Nineteen RT-PCR positive products were selected for cloning and sequencing based on the locality and time of their collection. PCR positive products were purified using a QIA Quick Purification Kit (Qiagen) and cloned into the pGEM-T Easy Vector System I (Promega, Madison, WI, USA). The plasmid clones were purified with a QIAprep Spin Miniprep Kit (Qiagen), and verified by digesting the plasmid DNA with *Eco*RI (New England Biolabs, UK) and separating it in a 1.2% agarose gel. Products were sequenced by Macrogen (Seoul, ROK). Sequences were deposited in GenBank under the accession numbers JX018147-JX018168 and JX018131-JX018146.

Similarity among genetic sequences for JEV prM and E proteins analyzed as part of this study and those publically available on the GenBank database was assessed using the National Center for Biotechnology Information (NCBI, Bethesda, MD, USA) BLAST network service. Sequences were aligned using the Clustal W method in MegAlign version 7.1 (DNA-STAR, Madison, WI, USA) and compared to published sequences for JEV strains collected from human, porcine, mosquito, and unreported sources collected at locations in Asia and Oceania as available from the GenBank database using phylogenetic analyses. The geographical origin, source, year of detection/isolation, strain name, and GenBank accession numbers for sequences used in phylogenetic analyses are reported in Tables 1 and 2. Phylogenetic trees were generated using neighbor-joining algorithms and the Jukes and Cantor matrix. Support for topology was calculated using 1,000 bootstrap replications.

**Table 1:** Strains of Japanese encephalitis virus reported on GenBank and used in phylogenetic analysis of pre-membrane protein coding genes.

Genotype	Strain	Source of Virus	Geographical Origin	Collection Date	Accession No.
1	JX61	Pig Serum	China	2008	GU556217
1	JX66	Pig	China	2008	FJ179364
1	SX09S-01	Swine brain	China	2009	HQ893545
1	SH17M-07	NA^a^	China	2007	EU429297
1	K01-GN	NA^a^	Republic of Korea	2005^b^	AY965852
1	K01-JB	NA^a^	Republic of Korea	2005^b^	AY965850
1	K01-JN	NA^a^	Republic of Korea	2005^b^	AY965851
1	K94P05	NA^a^	Republic of Korea	1999^b^	AF045551
1	KV1899	NA^a^	Republic of Korea	2003^b^	AY316157
1	4790-85	*Homo sapiens*	Thailand	2009^b^	GQ902062
1	JEV-eq-Tottori	Horse cerebrum	Japan	2003	AB594829
1	JEV-sw-Mie-40	Swine serum	Japan	2004	AB241118
2	Bennett	*Homo sapiens*	Republic of Korea	1951	HQ223285
2	B-1381-85	Pig	Thailand	2009^b^	GQ902061
2	FU	Human serum	Australia	199 9^b^	AF217620
2	JKT654	Mosquito	Indonesia	1978	HQ223287
3	47	Cerebrospinal fluid	China	1950	JF706269
3	Beijing-1	Human brain	China	1949	JEVBEICG
3	CTS	Human brain	China	2003^b^	AY243814
3	G35	Mosquito	China	2003^b^	AY243815
3	GB30	*Murina aurata*	China	1997	FJ185037
3	JEV-NJ1	*Culex*	China	2009	HM234674
3	LYZ	Human brain	China	2003^b^	AY243818
3	P3	Human brain	China	2003^b^	AY243844
3	SA14-12-1-7	NA^a^	China	2001^b^	AF416457
3	SA14-14-2*	SA-14 derivate	China	1953	AF315119
3	YUNNAN0902	*Sus scrofa*	China	2009	JQ086763
3	CH2195	Na^a^	Taiwan	1994	AF030550
3	CJN-L1	NA^a^	Taiwan	2003^b^	AY303794
3	HVI	*Aedes albopictus*	Taiwan	1998^b^	AF098735
3	T1P1-L4	NA^a^	Taiwan	2003^b^	AY303792
3	Indonesia	NA^a^	Indonesia	1993^b^	JEU03692
3	Nakayama*	Human brain	Japan	1395	JEU03694
3	JaOH0566	NA^a^	Japan	1997	AY508813
3	CNU-LP2x	NA^a^	Republic of Korea	2009^b^	GQ199609
4	JKT6468	Mosquito	Indonesia	1968	AY184212
5	XZ0934	NA^a^	China	2009^c^	JF915894

a Not available

b Submitted date

c Published date.

* Vaccine strains that have been used in the Republic of Korea.

**Table 2:** Strains of Japanese encephalitis virus reported on GenBank and used in phylogenetic analysis of envelope protein coding genes.

Genotype	Strain	Source of Virus	Geographical Origin	Collection Date	Accession No.
1	SC04-15	*Culex tritaeniorhynchus*	China	2006^b^	DQ404091
1	LX10P-09	Cerebrospinal fluid	China	2009	HM204528
1	LY5P-09	Cerebrospinal fluid	China	2009	HM204530
1	XP174M0-08	*Culex tritaeniorhynchus*	China	2008	HM204527
1	B2239	Pig	Thailand	1984	JEU70391
1	P19Br	Human	Thailand	1982	JEU70416
1	M859	Mosquito	Cambodia	1967	JEU70410
1	K01-JB	Mosquito	Republic of Korea	2001	FJ938221
1	K96A07	Mosquito	Republic of Korea	1996	FJ938219
2	JKT1749	Mosquito	Indonesia	1979	JEU70405
2	WTP-70-22	Mosquito	Malaysia	1970	JEU70421
3	Beijing 1	Human brain	China	1949	JEU70389
3	CH2195	NA^a^	China	1994	JEU92644
3	FJ03-97	*Homo sapiens*	China	2006^b^	DQ404127
3	GZ04-43	*Culex* sp	China	2006^b^	DQ404113
3	HLJ08-01	Swine	China	2008	GQ495004
3	HLJ08-02	Swine	China	2008	GQ495005
3	SA14-14-2*	SA-14 derivate	China	1953	AF315119
3	SH04-10	*Culex tritaeniorhynchus*	China	2006^b^	DQ404107
3	YNDL04-1	*Culex tritaeniorhynchus*	China	2006^b^	DQ404137
3	Chiang Mai	Human	Thailand	1964	JEU70393
3	B18A	Mosquito	Japan	1978	JEU70390
3	Mie44-1	Mosquito	Japan	1969	JEU70411
3	Nakayama*	Human brain	Japan	1935	JEU70413
3	Osaka	Mosquito	Japan	1979	JEU70414
3	Sagiyama	Mosquito	Japan	1957	JEU70419
3	JaNAr0990	Mosquito	Japan	1990	AY427797
3	JaOH0566	Human brain	Japan	1966	JEU70399
3	JaOH3767	Human brain	Japan	1967	JEU70400
3	K83P34	Mosquito	Republic of Korea	1983	FJ938231
3	K84A071	Mosquito	Republic of Korea	1984	FJ938224
3	K87A071	Mosquito	Republic of Korea	1987	FJ938226
3	K88A071	Mosquito	Republic of Korea	1988	FJ938228
3	K94A071	Mosquito	Republic of Korea	1994	FJ938217
3	H49778	Human	Sri Lanka	1987	JEU70395
3	Indonesia	Mosquito	Indonesia	1996^b^	JEU70397
3	JKT6468	Mosquito	Indonesia	1981	JEU70407
3	826309	Human	India	1982	JEU70403
3	P20778	Human	India	1958	JEU70415
3	R53567	NA^a^	India	1996^b^	JEU70418
3	PhAn1242	Pig serum	Philippines	1984	JEU70417
3	VN118	Mosquito	Vietnam	1979	JEU70420
4	JKT7003	Mosquito	Indonesia	1981	JEU70408
4	JKT9092	Mosquito	Indonesia	1981	JEU70409
4	2372	Human	Thailand	1979	JEU70401
5	XZ0934	Mosquito	China	2009	JF915894

a Not available

b Submitted date

* Vaccine strains that have been used in the Republic of Korea.

Nucleotide sequence information for the E protein was translated for JEV strains identified in mosquitoes as part of this study and vaccine strains currently used in ROK. Deduced amino acid differences were identified among strains and between those detected in mosquitoes and those used in the national vaccination program.

## Results

### Detection of JEV from Mosquito Samples

A total of 6,328 culicine mosquitoes, representing 12 species from 5 genera, were captured at 6 localities in ROK from June through October 2010. The most frequently collected species was *Culex tritaeniorhynchus* (45.5%, *n* = 2,880), followed by *Aedes vexans nipponii* (33.0%, *n* = 2,091), *Cx. pipiens* (11.6%, *n* = 736), *Cx. bitaeniorhynchus* (5.4%, *n* = 344), *Ochlerotatus koreicus* (2.9%, *n* = 181), *Aedes albopictus* (1.0%, *n* = 66), *Armigeres subalbatus* (0.4%, *n* = 23), *Cx. orientalis* (<0.1%, *n* = 3), *Aedes lineatopennis* (<0.1%, *n* = 1), *Cx. inatomii* (<0.1%, *n* = 1), *Mansonia uniformis* (<0.1%, *n* = 1), and *Ochlerotatus nipponicus* (<0.1%, *n* = 1) (Table 3). A total of 34/371 pools (9.2%) tested positive for JEV (Table 3). JEV was most frequently identified in pools of *Cx. tritaeniorhynchus* (24.0%, 29/121 pools), followed by *Cx. pipiens* (6.3%, 4/64 pools) and *Cx. bitaeniorhynchus* (3.8%, 1/26 pools) (Table 3). All other culicine species tested negative for JEV (Table 3). MLE for the number of JEV RNA-positive mosquitoes per 1,000 individuals were 11.8, 5.6, and 2.8 for *Cx. tritaeniorhynchus*, *Cx. pipiens*, and *Cx. bitaeniorhynchus* (Table 3).

**Table 3:** Total number of culicine mosquitoes collected at 6 localities in four provinces in Republic of Korea in 2010 and number of Japanese encephalitis virus positive pools (up to 30 mosquitoes) as detected using RT-PCR.

Species	Total Number Tested(% of Total)	Pools Tested (% of Total)	Positive Pools (MLE)^a^
*Aedes albopictus*	66 (1.0)	15 (4.0)	0
*Aedes lineatopennis*	1 (<0.1)	1 (0.3)	0
*Aedes vexans nipponii*	2,091 (33.0)	106 (28.6)	0
*Armigeres subalbatus*	23 (0.4)	9 (2.4)	0
*Culex bitaeniorhynchus*	344 (5.4)	26 (7.0)	1 (2.8)
*Culex inatomii*	1 (<0.1)	1 (0.3)	0
*Culex orientalis*	3 (<0.1)	2 (0.5)	0
*Culex pipiens*	736 (11.6)	64 (17.3)	4 (5.6)
*Culex tritaeniorhynchus*	2,880 (45.5)	121 (32.6)	29 (11.8)
*Mansonia uniformis*	1 (<0.1)	1 (0.3)	0
*Ochlerotatus koreicus*	181 (2.9)	24 (6.5)	0
*Ochlerotatus nipponicus*	1 (<0.1)	1 (0.3)	0
Total	6,328 (100.0)	371	34 (5.8)

Up to 30 specimens of culicine mosquitoes were pooled based on the locality and time of their collection.

a Maximum likelihood estimation (MLE) = estimated number of viral RNA-positive mosquitoes per 1,000.

Although JEV-positive mosquitoes were identified at horse farms in Ilsan and Jeju, no JEV-positive mosquitoes were identified at horse farms in Busan, Jangsu, or Gwacheon (Fig. 2). Most of the JEV-positive mosquitoes were collected in Munsan at Warrior Base, a US military complex located 4 km from the DMZ separating North and South Korea. One mosquito species, *Cx. pipiens* (MLE = 4.4), was positive for JEV at Jeju; two species, *Cx. tritaeniorhynchus* (MLE = 4.7) and *Cx. pipiens* (MLE = 16.1), were positive for JEV at Ilsan; while three species, *Cx. tritaeniorhynchus* (MLE = 13.3), *Cx. pipiens* (MLE = 5.8), and *Cx. bitaeniorhynchus* (MLE = 2.8), were positive for JEV at Munsan.

### Sequence Similarity and Phylogenetic Analysis of the prM and E Genes

**Figure 3:**
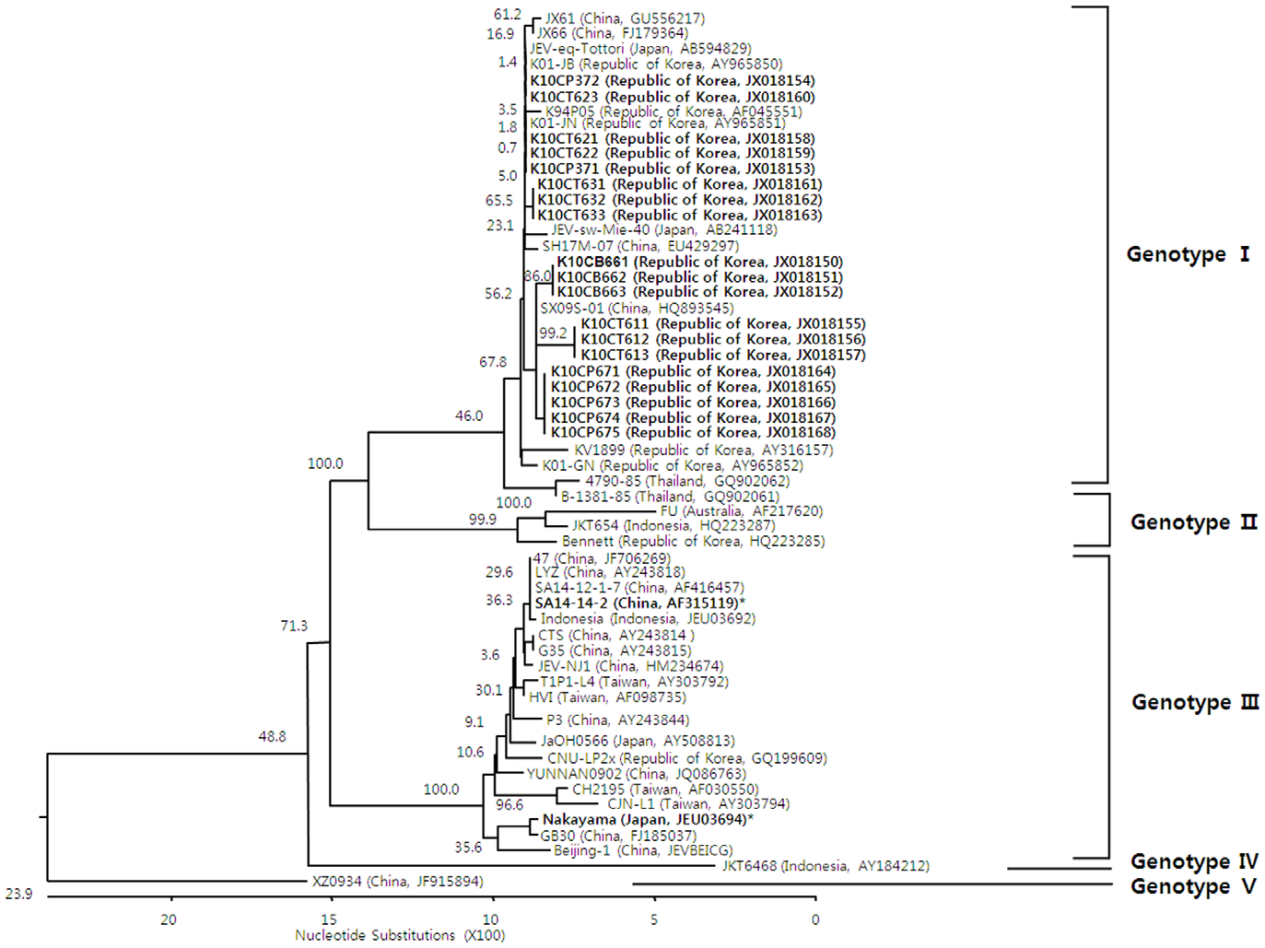
Phylogenetic tree illustrating the genetic relationship of nucleotide sequences for pre-membrane protein genes of Japanese encephalitis virus (JEV) strains identified in mosquitoes, Republic of Korea, 2010 (indicated in bold font) and reference sequences from other geographic regions as reported on GenBank. Genotypes of JEV strains are indicated on the right of the phylogenetic tree and were assigned according to Chen et al. [45], [46]. Bootstrap support values are shown. The scale bar indicates the number of mutations. Abbreviations for strains reported in this study are as follows: K10CT = Republic of Korea (ROK), 2010, *Culex tritaeniorhynchus*; K10CB = ROK, 2010, *Culex bitaeniorhynchus*; and K10CP = ROK, 2010, *Culex pipiens*. Vaccine strains that have been used in ROK are indicated in bold font and with an asterisk (*).

All nucleotide sequences for the prM protein coding region of JEV strains derived from the mosquitoes and sampled in ROK in the present study clustered with viruses previously classified as genotype I (Fig. 3). The prM protein coding nucleotide sequences from ROK analyzed in this study were 79.2–100% similar to other JEV strains isolated in China, Japan, Thailand, Taiwan, Indonesia, and Australia. Sequence similarity for prM protein coding sequences were 88.2–100% between JEV strains previously identified in ROK and those identified in this study.

**Figure 4:**
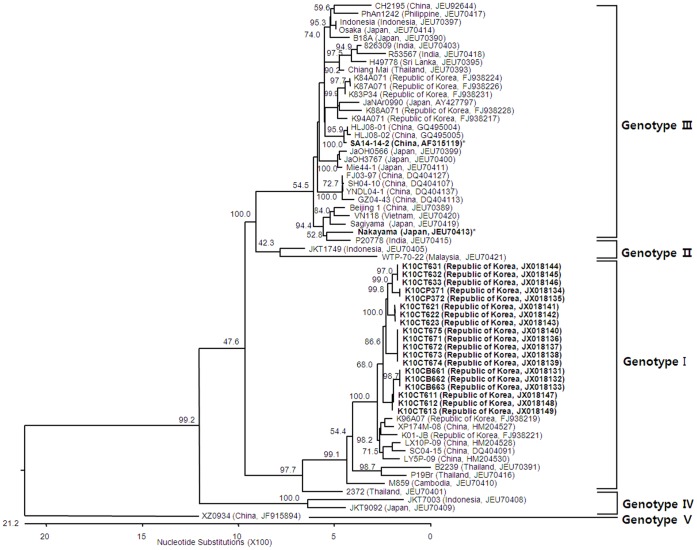
Phylogenetic tree illustrating the genetic relationship of nucleotide sequences for envelope protein coding genes of Japanese encephalitis virus (JEV) strains identified in mosquitoes, Republic of Korea, 2010 (indicated in bold font) and reference sequences for JEV strains from other geographic regions as reported on GenBank. Genotypes of JEV strains are indicated on the right of the phylogenetic tree and were assigned according to Chen et al. [45], [46]. Bootstrap support values are shown. The scale bar indicates the number of mutations. Abbreviations for strains reported in this study are as follows: K10CT = Republic of Korea (ROK), 2010, *Culex tritaeniorhynchus*; K10CB = ROK, 2010, *Culex bitaeniorhynchus*; and K10CP = ROK, 2010, *Culex pipiens*. Vaccine strains that have been used in ROK are indicated in bold font and with an asterisk (*).

Nucleotide sequences for the E protein coding region of JEV strains from mosquitoes sampled in ROK also formed clustered with genotype I viruses (Fig. 4). Nucleotide sequence similarity between the E protein coding region of viruses identified in this study and previously published sequences was 76.9–99.1%. Similarity of the E protein coding region was 87.5–99.1% when considering only strains of JEV originating from ROK.

### Comparison between JEV Strains in Mosquitoes and Vaccine Strains Currently Used in ROK

JEV strains identified in this study had 87.9–88.7% nucleotide sequence similarity and 96.5–97.9% amino acid sequence similarity compared to vaccine strain SA14-14-2 (China, AF315119) currently used in ROK based on analysis of prM protein coding region sequences. Nucleotide and amino acid sequence similarity were 87.5–88.7% and 94.3–95.7% when comparing prM sequences for JEV strains in mosquitoes with another vaccine strain used in ROK, Nakayama (Japan, JEU03694). E protein coding region sequences of JEV strains identified in this study were 87.6–88.0% and 96.4–97.2% similar with regard to nucleotide and amino acid similarity when compared with vaccine strain SA14-14-2. Nucleotide and amino acid sequence similarity was 87.6–88.0% and 96.4–97.2%, respectively, when comparing E protein coding regions of strains from mosquitoes with strain JEV Nakayama. Five amino acid residues of JEV strains identified in mosquitoes were different from vaccine strains used in ROK: E129 (Thr→Met), E176 (Val, Thr→Ile ), E222 (Arg→Ser, Pro), E327 (Ser→Thr), and E366 (Arg→Ser) (Table 4).

**Table 4:** Comparison of amino acids differences in the envelope protein between the Japanese encephalitis vaccine strains that have been used in Republic of Korea and those identified in mosquitoes for this study.

Strain	E107	E123	E129	E138	E176	E177	E222	E264	E279	E315	E327	E346	E366	E439
SA-14-14-2*	F	S	T	K	V	A	A	H	M	V	S	N	A	R
Nakayama*	L	S	T	E	T	T	A	Q	K	A	S	N	A	K
K10CP371	L	S	M	E	I	T	P	Q	K	A	T	N	S	K
K10CP372	L	S	M	E	I	T	S	Q	K	A	T	S	S	K
K10CT611	L	S	M	E	I	T	S	Q	K	A	T	S	S	K
K10CT612	L	S	M	E	I	T	S	Q	K	A	T	N	S	K
K10CT613	L	S	M	E	I	T	S	Q	K	A	T	N	S	K
K10CT621	L	S	M	E	I	T	S	Q	K	A	T	N	S	K
K10CT622	L	S	M	E	I	T	S	Q	K	A	T	N	S	K
K10CT623	L	S	M	E	I	T	S	Q	K	A	T	N	S	K
K10CT631	L	S	M	E	I	T	S	Q	K	A	T	S	S	K
K10CT632	L	S	M	E	I	T	S	Q	K	A	T	S	S	K
K10CT633	L	S	M	E	I	T	S	Q	K	A	T	S	S	K
K10CB661	L	S	M	E	I	T	S	Q	E	A	T	N	S	K
K10CB662	L	S	M	E	I	T	S	Q	E	A	T	N	S	K
K10CB663	L	S	M	E	I	T	S	Q	E	A	T	N	S	K
K10CP671	L	S	M	E	I	T	S	Q	K	A	T	N	S	K
K10CP672	L	S	M	E	I	T	S	Q	K	A	T	N	S	K
K10CP673	L	S	M	E	I	T	S	Q	K	A	T	N	S	K
K10CP674	L	S	M	E	I	T	S	Q	K	A	T	N	S	K
K10CP675	L	S	M	E	I	T	S	Q	K	A	T	N	S	K

Abbreviations: A, alanine; E, glutamic acid; F, phenylalanine; H, histidine; I, isoleucine; L, leucine; M. methionine; N, asparagines; P, proline; Q, glutamine; R, arginine; S, serine; T, threonine; V, valine. K10CT = Republic of Korea (ROK), 2010, *Culex tritaeniorhynchus*; K10CB = ROK, 2010, *Culex bitaeniorhynchus*; and K10CP = ROK, 2010, *Culex pipiens*.

* Vaccine strains that have been used in ROK.

## Discussion

JEV vector surveillance provides information regarding the distribution, intensity, and abundance of circulating viruses that can be used for the development and implementation of disease mitigation strategies by public health officials. As part of surveillance activities, it is important that mosquitoes be processed properly so that arboviruses can be identified in the laboratory. Mosquitoes that were trapped over a 24-hr period were transported to the 5^th^ Medical Detachment for identification and maintained at −70°C. Although many of the mosquitoes arrived dead, it has been shown that JEV RNA is stable up to 14 days even under relatively harsh conditions [24]. Thus, methodology for sampling and transport should have ensured accurate results.

The mandatory childhood immunization policy initiated in ROK in 1967 and fully implemented in 1971 greatly decreased the incidence of reported JE cases from epidemic proportions, which often exceeded 1,000 cases prior to 1982. From 1984 through 2009, there were no JEV outbreaks in the Korean population. During this same period, outbreaks/epidemics continued to be reported in India, China, and other countries that did not have comprehensive JEV vaccination programs. In 2010, an outbreak of 26 JE cases, including 7 deaths (26.9%), was reported in ROK [19]. This number likely under-represents the actual number of cases, as only severe cases of encephalitis lead to hospitalization and proper evaluation of JEV infection. Previous JEV vector surveillance programs in ROK have been limited and therefore, although it cannot be empirically evaluated, it is hypothesized that the extended rainy season through mid-September is believed to be responsible for large *Cx. tritaeniorhynchus* populations, the primary JEV vector in ROK. Additionally, an extended rainy season may have led to increased populations of potential secondary vectors, e.g., *Cx. pipiens* and *Cx. bitaeniorhynchus*. Phylogenetic analyses of JEV strains circulating in East Asia indicate that genotype I strains detected in mosquitoes have a relatively distant genetic relationship to genotype III vaccine strains currently used in ROK (Figs. 3 and 4). Additionally, five amino acid residues of JEV strains identified in mosquitoes were different from vaccine strains (Table 4). These data support the circulation of genetically divergent JEV strains in ROK during 2010 as compared to vaccine strains. Howver, there are evidences for partial protection by antibodies that cross-react within the JE serocomplex group of viruses [25]–[27] and therefore vaccine breakthrough may be an insufficient for an alternative explanation for the 2010 outbreak of JE in ROK. A detailed epidemiological analysis of the outbreak may have provided more clues on the underlying factors contributing to the outbreak although this was outside the scope of the current study.

*Cx. tritaeniorhynchus* is well known to be the primary vector for JEV in ROK and throughout much of Asia. However, in India and other parts of Asia, other *Culex spp.* are primary (e.g., *Cx. vishnui*) or secondary vectors (e.g., *Cx. pipiens* and *Cx. bitaeniorhynchus*) [28]–[32]. In this study, *Cx. tritaeniorhynchus* accounted for 85.3% of the JEV-positive pools of culicines while only comprising 32.6% of those tested (Table 3). Maximum likelihood methods estimate 11.8 JEV-positive individuals per 1,000 mosquitos sampled for this species (Table 3). Thus, these data indicate that *Cx. tritaeniorhynchus* carried JEV at relatively high rates in ROK during the period of the 2010 outbreak and therefore may have contributed to transmission of viruses at this location and time.

Both *Cx. tritaeniorhynchus* and *Cx. bitaeniorhynchus* are associated with rice paddies/water impoundments associated with large water birds, while *Cx. pipiens* is often associated with swine farms. More than one sub-species of *Cx. pipiens* is found in ROK. *Cx. pipiens molestus* is autogenous and occurs year-round, whereas *Cx. pipiens pallens* is not collected during the winter season in ROK. The taxonomy of these species has not been resolved and thus are reported as *Cx. pipiens* herein. JEV-positive *Cx. pipiens* were observed in 11.8% of PCR-positive pools while accounting for 17.3% of culicine pools tested (Table 3). Maximum likelihood methods estimated 5.6 JEV-positive individuals per 1,000 mosquitos sampled for this species (Table 3). Laboratory studies have demonstrated low vector competence for *Cx. pipiens* and therefore this species may be more likely to be a secondary vector when large populations of mosquitoes are present near pig farms [33]–[35]. In the early 1970s, JEV was isolated during the winter on two occasions from *Cx. pipiens* in ROK [36] which was somewhat surprising as this virus had been identified infrequently in this mosquito species in northern Asia. The role of *Cx. pipiens* in the transmission of JEV is not fully understood, but this species may contribute to virus spread in urban environments. Thus, *Cx. pipiens* may also have contributed to the JE outbreak in ROK during 2010.

*Cx. tritaeniorhynchus*, *Cx. bitaeniorhynchus*, and *Cx. pipiens* (a vector of West Nile virus in the United States) readily feed on birds and mammals, including man. *Cx. bitaeniorhynchus* accounted for 2.9% of JEV PCR-positive culicine pools detected in this study (Table 3). Maximum likelihood methods estimated only 2.8 JEV-positive individuals per 1,000 sampled (Table 3). Although JEV has been identified in *Cx. bitaeniorhynchus* in India and other countries, this species has only recently been implicated as a potential vector in ROK [37]. Therefore the role of this species in the maintenance and transmission of JEV in ROK is unknown.

Until the latter part of the 20th century, studies of JEV indicated that the predominance of JEV strains detected worldwide could be assigned to genotype III. Since then, there have been reports of JEV genotype I displacing genotype III in many regions [1], [14], [38]–[43], and genotype I is now recognized as the dominant strain in many areas. Although JEV genotype V has recently re-emerged in Asia (China and ROK) after more than a half-century [44], only genotype I viruses have been reported to be circulating in ROK during 1994–2009 [12]. Similarly, all nucleotide sequences for prM and E genes isolated from viruses obtained from mosquitoes and analyzed in this study phylogenetically clustered with JEV genotype I strains. Nucleotide and amino acid similarity results suggest that viruses identified in mosquitoes in the present study were more closely related to JEV strains circulating throughout Asian countries than vaccine strains currently used in ROK (Figs. 3 and 4; Table 4).

Results from this study demonstrate the utility of vector screening for surveillance of JEV in ROK. Additional studies that measure the impact of vectors (e.g., bionomics and vector competence) in the transmission of JEV and that incorporate environmental factors (e.g., weekly rainfall) are needed to define the roles of *Culex* species in the viral pathogenesis during outbreak and non-outbreak years. Furthermore, long-term longitudinal vector surveillance is necessary to better understand the dynamics of JEV transmission in ROK and to characterize the role of potential secondary vectors, e.g., *Cx. pipiens* and *Cx. bitaeniorhynchus*, in the maintenance and human transmission of JEV.
